# The Coming Physiology: A Prediction

**Published:** 1902-12

**Authors:** S. B. Palmer

**Affiliations:** Syracuse, N. Y.


### THE COMING PHYSIOLOGY: A PREDICTION.[1]

BY DR. S. B. PALMER, SYRACUSE, N. Y.

### FIRST CHAPTER.

Gentlemen,—By the kind words spoken here to-night I am assured that my life has been spared to live down the so-called heresies of the New Departure, for which I am very thankful. Life is too short to engage in another departure; and yet by the eye of faith I can read the preface of the coming physiology, something like this. Modern physiology teaches that life is energy, emanating from the Creator, which is the soul or spirit of matter. It is manifested in all matter, and may be recognized when matter in evolution has been raised from the elements into conditions to maintain life. Life is visible in vegetation, and in the lower animals, ascending to the highest creation, man. Electricity, as has been taught in

---

[1] Read at the banquet given in honor of Dr. S. B. Palmer, New York, October, 1902.

physics, has been the agent to unite the elements to form masses, to prepare for vegetable life.

### SECOND CHAPTER.

Life appears, as seen in the vegetable kingdom, and in addition, there is instinct manifesting guidance to seek moisture through the rootlets; also life and light through the foliage. This teaches co-operation between the soil and sunlight. The communication is through the sap, which to the tree or vine is as the blood to the animal; not only that, it is an electrical conductor between the positive pole, the foliage, and the roots, which are negative. Cut off the tallest vine at the root, and it withers and dies directly. Thus electricity has undergone evolution, and is adapted to the growth and needs of vegetation. The florist knows that flowers undergo civilization by proper cultivation and fall back by neglect.

### THIRD CHAPTER.

The third chapter teaches that the "clay," so to speak, of which man is made, is of a finer quality. Added to instinct, man has mind, will, thought, and reason. In past works on physiology there seems to be no scientific connection between those attributes and the body, nor had science advanced to a degree which could afford demonstration. What has been understood as the "electro-chemical theory" has been taken as the basis of this work,—namely:

Man is considered an organic living dynamo, the system being run by electrical currents, which are mainly generated from positive and negative food elements taken into the stomach. The stomach is a vital cell; the elements are organic, and by the process formerly called "digestion" pass through the process taking place in a fluid cell of zinc, copper, or carbon, in appropriate fluids, in the laboratory. Currents thus generated we call physical electricity. The electricity which is generated in a living cell from organic elements is called vital or organized electricity. Mind, will, etc., are phases of electricity. Life as mentioned is energy back of our knowledge. We see its manifestation in man, which is the most convincing phase of mental electricity of any hitherto set forth. For instance, consider brain and muscle storage batteries for vital electricity, and each organ a station commissioned to do its work under direction of life's agent, electricity. Thus, in dentistry, the deciduous teeth conform to the side of the jaws; later on the roots are absorbed and the permanent tooth takes its place. This process seems simple when it is considered electrically. Life directs the deposit to build up, and reverses the current to absorb the roots. Each process is conducted in time and in order, as teeth are in pairs, and adapted to their position in the jaws.

### FOURTH CHAPTER.

The fourth chapter teaches the relation of mind to matter, which is demonstrated by electricity generated from organized elements. In telegraphing the mind directs the fingers to manipulate the key by which symbols of words are put in circuit to a receiving operator, who translates and delivers the message. This illustrates nothing different from writing letters except the mode of conveyance. The invention of the telephone, aside from the uses to which it was designed, has furnished demonstrations which warrant the introduction of the teachings to be found in this new physiology; that is, mind, thought, will, etc., are phases of intellectual electricity that relate to the body and belong to science for discussion, as truly physiological or mental science has done hitherto. This work teaches that the gulf between physical science and mental science has been bridged over. It does not include metaphysics proper, but science in connection with thought, will, and reason, relating to present life here and now. As an illustration: It is well understood that the voice of a speaker is borne on the air by wave-sounds that are set in motion by the vocal organs. Also that the voice cannot be heard at any great distance. The vocal organs are under mental control and direction through life's energy, conveyed to the organs by vital electricity. The process seems simple by which vital electricity is converted into physical electricity, and again reconverted into speech, even in the voice of the transmitter. The waves of sound strike a disk, which in effect corresponds with a telegraph key, though with inconceivable rapidity. Thus the impressions are carried forward with lightning velocity at the receiving end of the line, by a receiver, and speech is restored. Mental electricity inspired the vocal organs to do life's bidding; by physical electricity the message is carried and delivered in the tone and spirit of the author of the message. Wireless telegraphy teaches not only the same principle, but an advance lesson in inspiration, and man's relation to Divinity. This doctrine does not strictly belong to dentistry, but is predicted as belonging to science of life in life.

Science is truth, and divine. It will, in the Creator's good time, do away with fallible traditions and inspirations. One great hinderance to the world's progress is looking backward for light and guidance instead of forward. Inspiration is open to man now as it has ever been. When individuals can have faith in the Divinity within, and more courage to think for themselves, they will hold to all truths vouchsafed in the past, and as a foundation upon which to stand and receive new light, and new truths, that Divinity offers to all mankind who can reverently trust the conditions upon which they are offered. The trend of investigation indicates that the discovery may soon be announced that electricity is an element which is the connecting medium between mind and matter, or between man and his Maker. Such discovery would solve the mystery which at present shrouds electricity, and it would stimulate man to come into closer communion with his Maker.

